# CT perfusion imaging of the liver and the spleen in patients with cirrhosis: Is there a correlation between perfusion and portal venous hypertension?

**DOI:** 10.1007/s00330-017-4788-x

**Published:** 2017-03-20

**Authors:** Emina Talakić, Silvia Schaffellner, Daniela Kniepeiss, Helmut Mueller, Rudolf Stauber, Franz Quehenberger, Helmut Schoellnast

**Affiliations:** 1 Division of General Radiology, Department of Radiology, Medical University of Graz, Auenbruggerplatz 9, Graz, A-8036 Austria; 2 Department of Surgery, Division of Transplantation Surgery, Medical University of Graz, Graz, Austria; 3 Department of Internal Medicine, Division of Gastoenterology and Hepatology, Medical University of Graz, Auenbruggerplatz 15, 8036 Graz, Austria; 4 Institute for Medical Informatics, Statistics and Documentation, Medical University of Graz, Auenbruggerplatz 2, Graz, 8036 Austria

**Keywords:** CT-perfusion, Liver, Spleen, Cirrhosis, Portal hypertension

## Abstract

**Objectives:**

To correlate hepatic and splenic CT perfusion parameters with hepatic venous pressure gradient (HVPG) measurements in patients with cirrhosis.

**Methods:**

Twenty-one patients with cirrhosis (males, 17; females, 4; mean ± SD age, 57 ± 7 years) underwent hepatic and splenic perfusion CT on a 320-detector row volume scanner as well as invasive measurement of HVPG. Different CT perfusion algorithms (maximum slope analysis and Patlak plot) were used to measure hepatic arterial flow (HAF), portal venous flow (PVF), hepatic perfusion index (HPI), splenic arterial flow (SAF), splenic blood volume (SBV) and splenic clearance (SCL). Hepatic and splenic perfusion parameters were correlated with HVPG, and sensitivity and specificity for detection of severe portal hypertension (≥12 mmHg) were calculated.

**Results:**

The Spearman correlation coefficient was −0.53 (p < 0.05) between SAF and HVPG, and −0.68 (p < 0.01) between HVPG and SCL. Using a cut-off value of 125 ml/min/100 ml for SCL, sensitivity for detection of a HVPG of ≥12 mmHg was 94%, and specificity 100%. There was no significant correlation between hepatic perfusion parameters and HVPG.

**Conclusion:**

CT perfusion in patients with cirrhosis showed a strong correlation between SCL and HVPG and may be used for detection of severe portal hypertension.

***Key points*:**

• *SAF and SCL are statistically significantly correlated with HVPG*

• *SCL showed stronger correlation with HVPG than SAF*

• *125 ml*/*min*/*100 ml SCL*-*cut*-*off yielded 94* % *sensitivity*, *100* % *specificity for severe PH*

• *HAF*, *PVF and HPI showed no statistically significant correlation with HVPG*

## Introduction

Portal hypertension (PH) is defined as an increase in the pressure in the portal vein and its territory, and is one of the main causes of severe complications and death in patients with cirrhosis and, therefore, the main prognostic factor in cirrhosis [1]. In normal fasted subjects at rest and in the supine position, portal pressure ranges from 7 to 12 mmHg [2]. The direct measurement of portal pressure is a markedly invasive technique that is no longer performed in patients with cirrhosis; the indirect, less invasive technique of measuring the hepatic venous pressure gradient (HVPG) is used as a standard of reference to estimate the severity of PH in cirrhosis. In healthy subjects, the pressure gradient between the portal vein territory and the vena cava territory ranges from 1 to 4 mmHg [3]. PH is considered moderate when the HVPG ranges from 5 to 10 mmHg and severe when the HVPG is greater than 10–12 mmHg. [1, 4, 5]. An HVPG threshold of 10 mmHg is termed clinically significant PH as it predicts the risk of complications such as formation of oesophageal varices and development of hepatocellular carcinoma [6–8]. An HVPG >12 mmHg is associated with the risk of variceal bleeding [4] and an HVPG >16 mmHg correlates with increased mortality [9, 10].

Although HVPG measurement is a safe technique with only minor complications in <1% of patients [1], non-invasive techniques for HVPG assessment would be more favourable than invasive techniques. Computed tomography perfusion imaging (CTP) is a functional imaging technique that has been proven to allow quantification of the hepatic blood circulation in patients with diffuse liver disease [11–13]. As the hepatic blood circulation is commonly affected by chronic liver disease, mainly due to parenchymal remodeling resulting in an increased vascular resistance of the liver, CTP may be a valuable tool for noninvasive assessment of PH. In addition, splenic blood circulation assessed by CTP may be influenced by PH.

The aim of this study was to evaluate whether a correlation exists between hepatic and splenic perfusion parameters assessed by CTP and HVPG assessed by hepatic venous catherization.

## Materials and methods

### Patient population

This prospective study was approved by the Institutional Ethics Committee and informed consent was obtained from all patients. Patients who underwent HVPG measurement for evaluation of liver transplantation underwent CTP of the liver and the spleen. Inclusion criteria were: (a) HVPG measurement for suspected portal hypertension, and (b) age 18 years or older. Exclusion criteria were: (a) renal impairment (estimated glomerular filtration rate (eGFR) <45 ml/min/1,73 qm), (b) other contraindications to the application of iodinated contrast media, and (c) pregnancy.

Twenty-one patients (18 male, three female; mean ± SD age, 57 ± 7 years; range, 41–69 years) were included in the study. Twenty patients had cirrhosis and one patient had neuroendocrine liver metastases. Of the patients with cirrhosis, Child-Pugh classification was A in two patients, B in eight patients and C in eight patients. In two patients no classification was available in the medical records. Fourteen patients had alcoholic cirrhosis, three patients had cirrhosis due to hepatitis C and three patients had cryptogenic cirrhosis.

All patients underwent CTP of the liver and the spleen as well as HVPG measurement. The mean period (±SD) between CTP and HPGV measurement was 2 ± 2 days (range, 0–9 days).

### CTP imaging technique

All VCTP studies were performed using a 320-detector row CT (Aquilion One, Toshiba Medical Systems, Otawara, Japan) following an overnight fast. Scanning parameters were as follows: 100 kV tube voltage, 100 mA tube current, 0.5 s gantry rotation time and 320 x 0.5-mm section thickness. The detector width was 16 cm. A low-dose pre-contrast helical scan of the abdomen during suspended respiration following inspiration was performed to locate the liver and the spleen and to plan the scan position for the CTP study. As recommended for CT-angiography, contrast dose and injection rate were adjusted to patients’ body weight in our CT-perfusion protocol [14] (40–60 ml: 50–69 kg body weight, 40 ml; 70–89 kg body weight, 50 ml; and >90 kg body weight, 60 ml).Iomeprol 400 mgI/ml (Iomeron®, Bracco, Milano, Italy) was injected at a flow rate of 6–8 ml/s (50–69 kg body weight, 6 ml/s; 70–89 kg body weight, 7 ml/s; and >90 kg body weight, 8 ml/s), followed by the same volume of saline solution at the same injection rate using a dual-head power injector (Ulrich Medical, Chesterfield, MO, USA). All in all 21 volume acquisitions were obtained without table movement in all patients. After acquisition of two pre-contrast volumes, eight volumes were acquired during the early period of the injection protocol with an interscan gap of 1 s followed by seven volume acquisitions with an interscan gap of 2 s and by further four volume acquisitions with an interscan gap of 4 s. Total scan time was 80 s. Every patient was instructed to breathe normally during the examination and to avoid deep and irregular breathing. A band compressing the upper abdomen was used to reduce liver excursions. The total dose-length product (DLP) of 21 volumes was 1,031.1 mGy.cm, which is equivalent to an effective dose of 15.4 mSv (k 0.015).

### Image analysis

Post-processing was performed using body perfusion software (Body Perfusion, Toshiba Medical Systems) as available on the CT equipment. First, body registration to correct motion between the dynamic volumes was performed. Following registration, the corrected volumes were loaded into the body perfusion software.

**Fig. 1:**
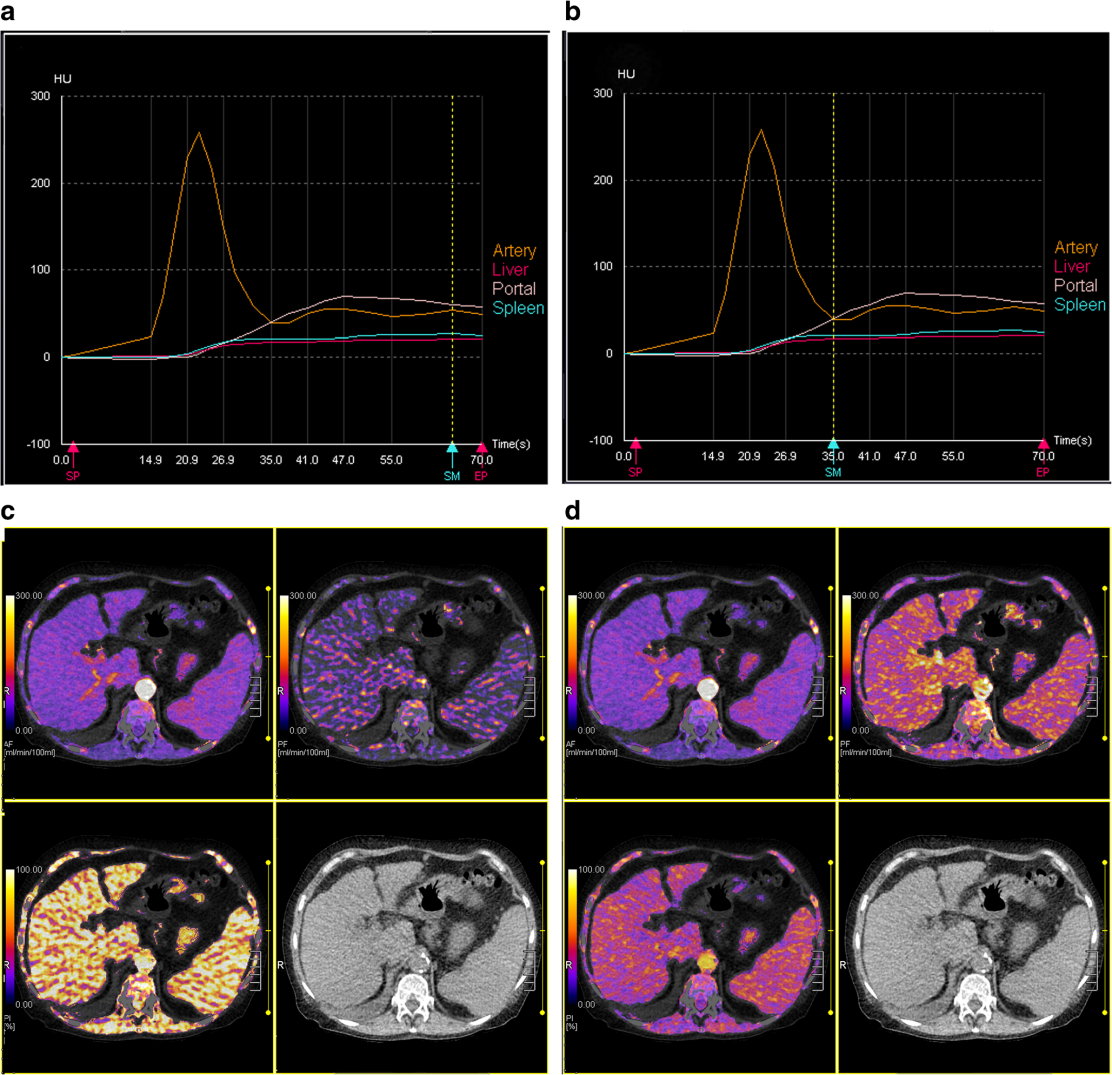
(**A**–**D**) Dual input maximum slope model of the liver yielding time-density-curves (TDC) for the aorta, the portal vein, the spleen and the liver (**A**, **B**) and parametric images (**C**, **D**) for hepatic arterial flow (HAF; left top), portal venous flow (PVF; right top) and hepatic perfusion index (HPI; left bottom) as well as corresponding pre-contrast grey-scale images (right bottom). The peak point of the TDC of the spleen is used to separate the hepatic artery circulation before the peak point and the portal vein circulation after the peak point, respectively. In patients with cirrhosis, the TDC of the liver may be flattened with a delayed peak (**A**). Perfusion calculation with this setting led to very low PVF and very high HPI (**C**). Manual adaptation with placement of the breakpoint at the crossing point of the aortal and portal venous TDC (**B**) led to significantly higher PVF and lower HPI (**D**). HAF, PVF and HPI in this patient were 68.5 ml/min/100 ml, 51.2 ml/min/100 ml and 61.4% for the standard setting (**A**, **C**), and 71.4 ml/min/100 ml, 126.5 ml/min/100 ml and 38.1% for the manual adapted setting (**B**, **D**). Severe portal hypertension (HVPG 22 mmHg) may contribute to the flat shape of the splenic TDC in this patient. *SP* start point, *EP* end point, *SM* spleen maximum

The dual input maximum slope model [15] was used for perfusion analysis of the liver. Two methods were used with regard to the settings of the regions of interest (ROIs) in all patients. In Method I the ROIs were manually placed in the abdominal aorta, the portal vein, the spleen and normal liver parenchyma to generate respective time-density curves (TDCs). The generated TDCs represented the hepatic artery input function and the portal vein input function, respectively (Fig. 1A). The peak point of the generated TDC of the spleen is used to separate the hepatic artery circulation before the peak point and the portal vein circulation after the peak point, respectively. In Method II the break point was manually set at the crossing point of the aortal and portal venous TDC (Fig. 1B). This adapted method was performed as it has been shown that in patients with portal venous hypertension peak splenic enhancement may be delayed, which may alter perfusion measurements due to inaccurate separation of arterial and portal venous perfusion [16]. Hepatic arterial blood flow (HAF; ml/min/100 ml), portal venous blood flow (PVF; ml/min/100 ml), as well as the hepatic perfusion index (HPI; HAF/(HAF + PVF); %) were calculated for all liver segments and the results were averaged for the whole liver.

The single input maximum slope and the Patlak plot model [15] were used for perfusion analysis of the spleen. ROIs were manually placed in the abdominal aorta and the spleen to generate respective TDCs. With the use of the single input maximum slope model the splenic arterial blood flow (SAF; ml/min/100 ml) and with the Patlak plot model, the splenic blood volume (SBV; ml/100 ml) and the splenic clearance (SCL; ml/min/100 ml), which is the total flux from the intravascular space to the extravascular space, were calculated.

In addition to CT-perfusion parameters, morphological features such as presence of perihepatic ascites, portosystemic collateral vessels and portal thrombosis were qualitatively assessed and spleen volume was quantitatively assessed using semiautomatic volumetric software (Vitrea, Toshiba Medical Systems).

### HVPG measurement

HVPG measurements were carried out according to established standards [17] following an overnight fast. Under ultrasound guidance, the right internal jugular vein was cannulated and a 7-French balloon catheter (Boston Scientific) was guided into the right hepatic vein for the measurement of wedged and free hepatic venous pressures. HVPG was calculated from the difference between wedged hepatic venous pressure (WHVP) and free hepatic venous pressure, and the mean of triplicate measurements was computed.

### Statistical analysis

The data were descriptively reviewed. The significances of differences in medians between Method I and Method II were analysed using Wilcoxon’s test. Spearman’s test was used for correlation of the perfusion parameters with the HVPG. Comparison of the parameters between the severe (≥12 mmHg) and moderate (<12 mmHg) portal hypertension groups and between patients with and without portal thrombosis was assessed using the Mann-Whitney test. Receiver operating characteristics (ROC) analysis was performed to calculate cut-off values for differentiation between moderate and severe portal hypertension. The significance of distribution of morphological features between the two groups was tested using the Chi-square test. Statistical analysis was performed with commercially available software (IBM SPSS Statistics, Version 23, SPSS Inc., Chicago, IL, USA). A P-value <0.05 was considered statistically significant.

## Results

Details on hepatic and splenic perfusion values and HVPG are listed in Table 1.

**Table 1:** Details of hepatic and splenic perfusion parameters, splenic volume and HVPG

		Median	IQR	Min	Max
Liver	*Method I*				
	HAF (ml/min/100 ml)	56.7	26.2	26.4	123.9
	PVF (ml/min/100 ml)	118.0	81.9	31.1	255.8
	HPI (%)	35.9	26.7	16.0	81.7
	*Method II*				
	HAF (ml/min/100 ml)	56.7	30.3	26.4	121.2
	PVF (ml/min/100 ml)	134.1	62.6	93.1	255.8
	HPI (%)	35.5	16.0	16.0	48.0
Spleen	SAF (ml/min/100 ml)	107.8	56.4	67.2	209.2
	SBV (ml/100 ml)	1.5	2.3	0.4	39.9
	SCL (ml/min/100 ml)	86,4	69	23.4	160,8
	V (ml)	577.1	435.0	149.4	1274.1
HVPG (mmHg)		14	9	2	28

*HAF* hepatic arterial flow, *PVF* portal venous flow, *HPI* perfusion index, *SAF* splenic arterial flow, *SBV* splenic blood volume, *SCL* splenic clearance, *V* volume, *HVPG* hepatic venous pressure gradient

There was no statistically significant difference between Method I and Method II in HAF, whereas PVF and HPI were statistically significant different (p < 0.05). The mean difference in medians (±SD) was 1.0 ± 2.3 ml/min/ml for HAF (range, 0–9.1 ml/min/100 ml), 22.6 ± 43.3 ml/min/100 ml for PVF (range, 0–146.1 ml/min/100 ml) and 9.1 ± 14.4 ml/min/100 ml for HPI (range, 0–41.6 ml/min/100 ml).

**Fig. 2:**
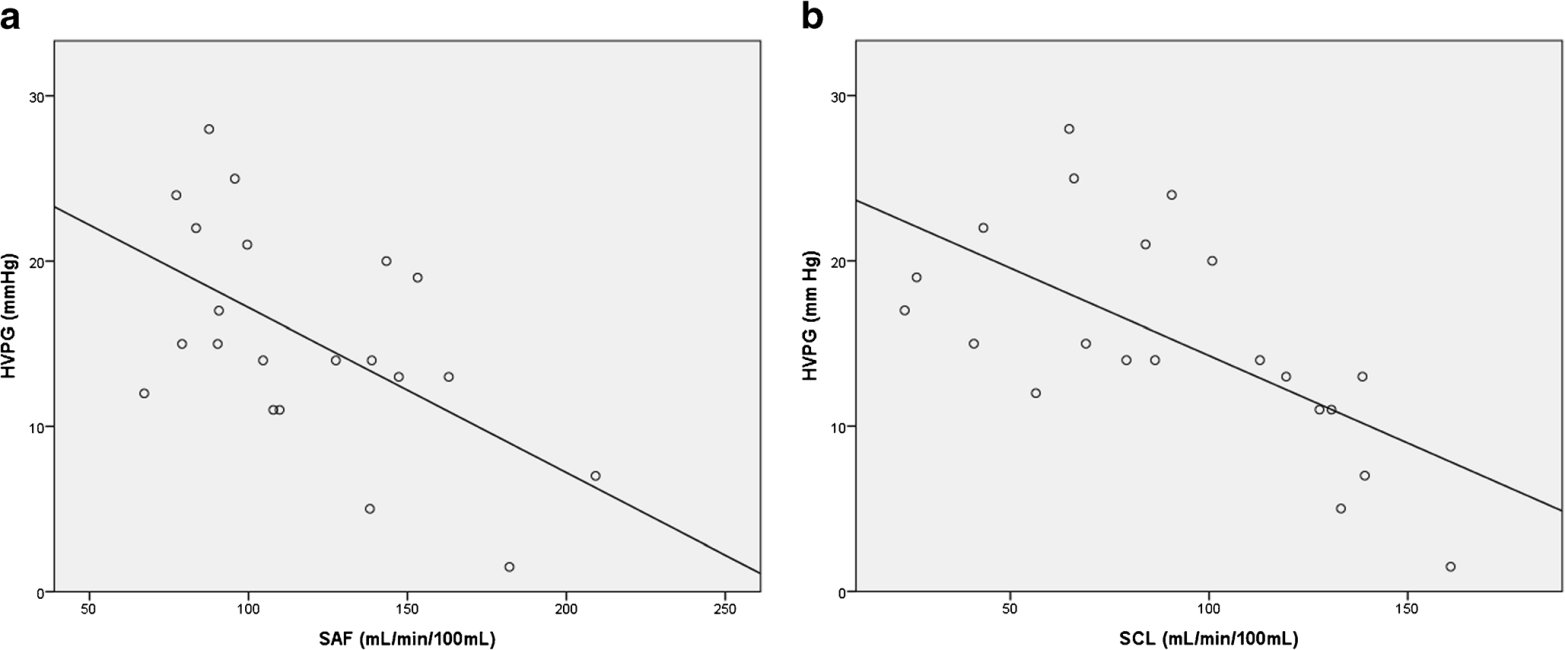
(**A**, **B**) Correlation between splenic arterial flow (SAF) and hepatovenous pressure gradient (HVPG) (**A**) and splenic clearance (SCL) and HVPG (**B**) showing a moderate negative correlation (r = 0.53. p < 0.05) for SAF and strong negative correlation (r = 0.68, p < 0.01) for SCL, respectively

For Method I as well as for Method II no statistically significant correlation was found between HAF, PVF, HPI and HVPG. On the contrary, SAF and SCL showed a statistically significant negative correlation with the HVPG, whereas SBV showed no correlation. The Spearman correlation coefficient was −0.53 (p < 0.05) for SAF and −0.68 (p < 0.01) for SCL. Figure 2 shows scatter plots for SAF and HVPG and for SCL and HVPG, respectively. There was no statistically significant correlation between splenic volume and HVPG.

Five patients had moderate (HVPG <12 mmHg) and 16 patients had severe (HVPG ≥12 mmHg) portal hypertension. The median HVPG was 7 mmHg (interquartile range (IQR), 8 mmHg; range, 2–11 mmHg) in the moderate portal hypertension group and 16 mmHg (IQR, 8 mmHg; range, 12–28 mmHg) in the severe portal hypertension group. Child-Pugh classification was A in one patient, B in two patients and C in one patient in the moderate portal hypertension group. One patient of this group had no cirrhosis. In the severe portal hypertension group Child-Pugh classification was A in one patient, B in six patients and C in seven patients. In two patients of this group Child-Pugh classification was not available. Body weight did not significantly differ between the two groups. Median body weight was 64 kg (range, 60–109 kg) in the moderate portal hypertension group and 81.5 kg (range, 60–110 kg) in the severe portal hypertension group.

SCL was statistically significantly different in patients with moderate and those with severe portal hypertension (P < 0.01), whereas SAF was not different between the two groups. Median SCL in patients with moderate portal hypertension was 133.2 ml/min/100 ml (IQR, 20.7; range, 127.8–160.8 ml/min/100 ml); in patients with severe portal hypertension median SCL was 74.1 ml/min/100 ml (IQR, 51.8, range, 23.4–138.6 ml/min/100 ml).

**Fig. 3:**
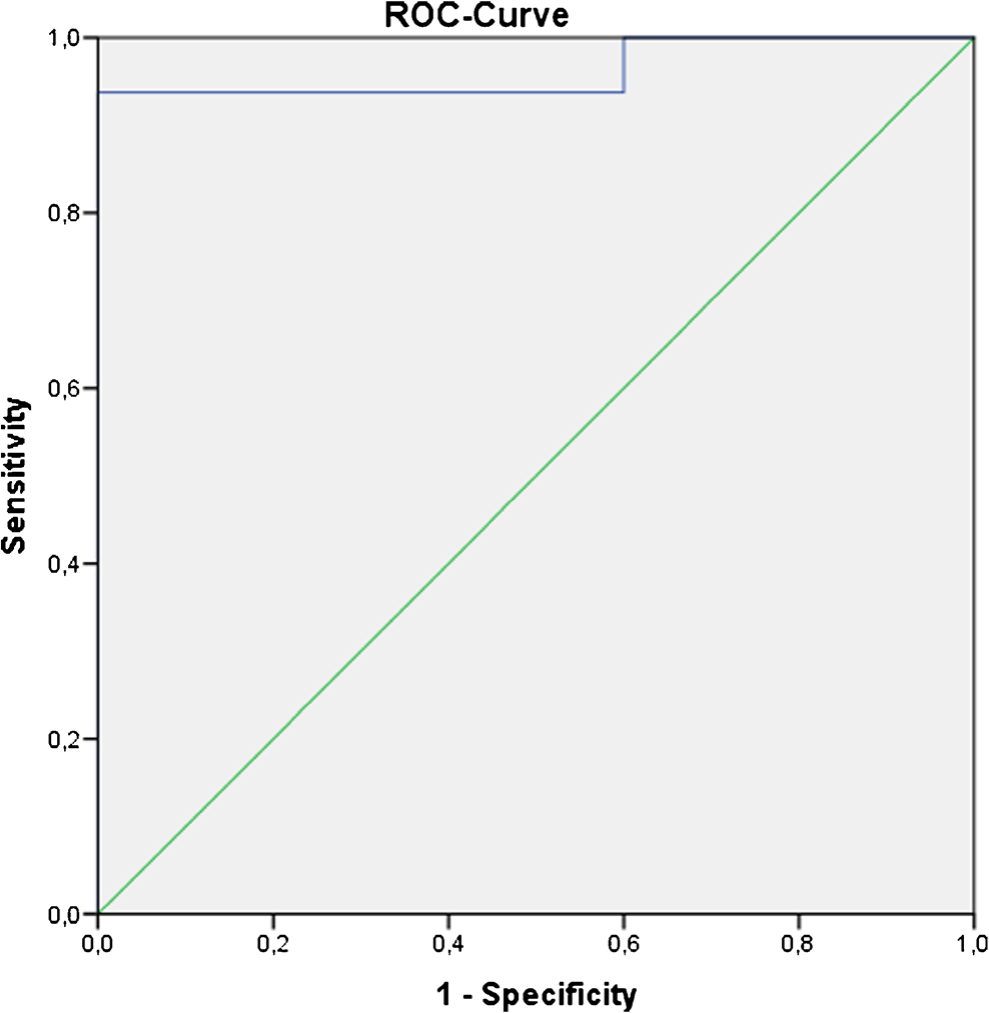
Receiver operating characteristic (ROC) curves of for differentiation between moderate and severe portal hypertension (PH) splenic clearance (SCL) for identifying severe PH. Area under the ROC was 0.96 (standard error, 0.04; 95% CI, 0.88–1)

ROC analysis of SCL for differentiation between moderate and severe portal hypertension showed an area under the curve of 0.96 with a standard error of 0.04 (95% confidence interval (CI), 0.88–1) (Fig. 3). Using a cut-off value of 125 ml/min/100 ml for SCL, sensitivity for detection of severe portal hypertension was 94% with a specificity of 100%.

Perihepatic ascites was present in three patients with moderate portal hypertension and in seven patients with severe portal hypertension; portal systemic varicose collateral vessels were present in three patients with moderate portal hypertension and in six patients with severe portal hypertension; and portal vein thrombosis was present in none of the patients with moderate portal hypertension and in four patients with severe portal hypertension. The distribution of these morphological parameters between patients with moderate and those with severe portal hypertension was not statistically significant. There was no statistically significant difference in splenic perfusion parameters and in HVPG between patients with and those without portal thrombosis. The median SAF was 93 ml/min/100 ml (IQR, 65.6; range, 77.3–163 ml/min/100 ml) in patients with portal thrombosis and 109.8 ml/min/100 ml (IQR, 56.2; range, 67.2–209.2 ml/min/100 ml) in patients without portal thrombosis. The corresponding values were 1.9 ml/100 ml (IQR, 1.4; range 1.2–2.7 ml/100 ml) and 1.5 ml/100 ml (IQR, 3.8; range, 0.4–39.9 ml/100 ml) for SBV, and 79.8 ml/min/100 ml (IQR, 59.8; range, 66–138.6 ml/min/100 ml) and 86.4 ml/min/100 ml (IQR, 79.5; range, 23.4–160.8 ml/min/100 ml) for SCL. Median HVPG was 19.5 mmHg (IQR, 11; range 13–25 mmHg) in patients with portal thrombosis, and 14 mmHg (IQR, 9; range, 2–28 mmHg) in those without portal thrombosis.

## Discussion

Our study results showed a strong negative correlation between SCL and HVPG and a moderate negative correlation between SAF and HVPG. Using a SCL cut-off value of 125 ml/min/100 ml sensitivity for detection of severe PH (≥12 mmHg) was 94%, and specificity was 100%. This indicates that perfusion CT of the spleen with calculation of the clearance which is the total flux of the contrast media from the intravascular space to the extravascular space in ml/min per 100 ml of tissue may be a non-invasive tool for estimation of the HVPG. We assume that a high portal venous pressure increases the pressure within the splenic interstitium which contradicts the flux of iodine from the intravascular space to the extravascular interstitial space. The correlation between SAF and HVPG is in accordance with the limited literature. In a study by Tsushima et al. [18], the authors reported a lower SAF in patients with chronic liver disease than in a control group and also found a significant negative correlation between SAF and WHVP in 11 patients. In contrast to our study, the correlation between SAF and WHVP was strong (r = 0.741, p = 0.0024), whereas in our study the correlation between SAF and HVPG was moderate. However, another study [19] failed to show a statistically significant difference in SAF between patients with and patients without cirrhosis, although a trend toward lower perfusion in patients with cirrhosis was observed. In a study by Sauter et al. [20] on dynamic contrast-enhanced splenic CT in patients with and without cirrhosis, splenic BV and K(trans), which is a parameter used in different perfusion software and which is comparable to SCL, were statistically significant different between the two groups, which is for K(trans) in accordance with our study. However, no correlation with HVPG was performed in the study by Sauter et al. [20].

The liver perfusion parameters did not correlate with HVPG and failed to separate patients with severe PH from patients with moderate PH. To the best of our knowledge, no data are available in the literature on hepatic CT perfusion in patients with PH. However, several studies assessed perfusion with scintigraphy and a clear reduction of portal perfusion was shown in patients with PH compared to healthy patients [21–23]. The sensitivity of scintigraphy in detecting PH, based on a portal contribution of ≤66%, was 62%, and specificity was 100%. Portal contribution to liver perfusion was statistically significantly negative correlated to HVPG (r = −0.43) [22]. We could not reproduce this finding in our CT perfusion study. Using contrast-enhanced ultrasonography, Jeong et al. [24] reported that the intrahepatic transit time, which is the time from hepatic arterial arrival to hepatic venous arrival, moderately correlated with HVPG (r = −0.613) and that this parameter was the most accurate one to diagnose severe PH (≥12 mmHg). Using a cut-off value of 6 s, sensitivity was 91% and 85%, and specificity was 89% and 78%, respectively, for the two reviewers. The false-positive rate was only 2%; however, the false-negative rates were 35% and 40%, respectively. With the software used in our study, the intrahepatic transit time was not available and this parameter could not be compared to the literature. In a recent study by Annet et al. [25] on hepatic flow parameters measured with MR imaging, HVPG was significantly correlated with all flow parameters. The correlation was substantial for the portal fraction (r = −0.769, p ≤ .001), the apparent portal perfusion (r = −0.726, p ≤ .001), and the mean transit time (r = 0.721, p ≤ .001). Weak but significant correlations were found for apparent arterial perfusion (r = 0.542, p ≤ .001), and distribution volume (r = 0.437, p ≤ .002). In contrast, no significant association was found between HVPG and the portal flow in a study by Gouya et al. [26], whereas the azygos flow and HVPG were significantly correlated. The different imaging techniques with different models of perfusion calculation may contribute to the inconsistent results.

Splenic volume was not significantly correlated to HVPG in our study. This is in contrast to a study by Kihira et al. [27] which showed such a correlation. However, the correlation coefficient was only weak (0.32) in that study. In a recent study by Pickhardt et al. [28] splenic volume allowed for non-invasive staging of hepatic fibrosis, but this finding may not be transferable to patients with cirrhosis and different levels of portal hypertension.

Using different ROI settings for separation between HAF and PVF led to significant differences in PVF and HPI calculation in our patients. This is in accordance with a study by Fischer et al. [16], which showed that peak splenic enhancement, which defines the break point between HAF and PVF, is delayed in patients with PH, resulting in a miscalculation of hepatic perfusion parameters. The authors recommended using peak renal enhancement instead of peak splenic enhancement in patients with PH. In our study, the break point was manually set at the crossing point of the aortal and portal venous TDC, which indicates the time point of replacement of the HAF by the PVF. We believe that this may be a more accurate method for hepatic blood flow separation than using a ROI within the renal parenchyma.

The small number of patients which may have influenced the results and overestimated sensitivity and specificity in detection of severe PH has to be considered as a limitation of this study. However, the standard error of the AUC in ROC analysis was only 0.04 and consequently a larger study cannot completely contradict our results. Only improvements of the estimates of the level of sensitivity and specificity will occur. Further studies are needed to confirm our first promising findings. The limitation of the results to the specific type of CT scanner, software, scan protocol and injection protocol used in this study has to be considered as further limitation of the study. Parameter values from our study cannot be transferred to CT perfusion using different models of perfusion calculation. In addition, other perfusion parameters such as mean transit time were not available in the used model and could not be assessed.

In conclusion, on CT-perfusion in patients with PH, SCL showed a strong correlation with HVPG and may be used as a parameter for detection of severe PH.

## Abbreviations and acronyms

HAF: Hepatic arterial flow (ml/min/100 ml)
HPI: Hepatic perfusion index (%)
HVPG: Hepatic venous pressure gradient (mmHg)
PH: Portal hypertension
PVF: Portal venous flow (ml/min/100 ml)
SAF: Splenic arterial flow (ml/min/100 ml)
SBV: Splenic blood volume (ml/100 ml)
SCL: Splenic clearance (ml/min/100 ml)
